# Two New Iridoids from *Verbena officinalis* L.

**DOI:** 10.3390/molecules190710473

**Published:** 2014-07-18

**Authors:** Jicheng Shu, Guixin Chou, Zhengtao Wang

**Affiliations:** 1 The MOE Key Laboratory for Standardization of Chinese Medicines and SATCM Key Laboratory for New Resources and Quality Evaluation of Chinese Medicines, Shanghai R&D Center for Standardization of Chinese Medicines, Institute of Chinese Materia Medica of Shanghai University of Traditional Chinese Medicine, Cai Lun Road 1200, Zhangjiang, Shanghai 201203, ChinaE-Mail: shujc210@163.com; 2 Key Laboratory of Modern Preparation of Traditional Chinese Medicines, Ministry of Education, Jiangxi University of Traditional Chinese Medicine, Nanchang 330004, China

**Keywords:** *Verbenaofficinalis* L., iridoids, spectroscopic methods

## Abstract

Two new iridoids, 3-(5-(methoxycarbonyl)-2-oxo-2*H*-pyran-3-yl)butanoic acid, named verbeofflin I (**1**), and 7-hydroxydehydrohastatoside (**2**), were isolated from the aerial part of *Verbena officinalis* L, along with three known iridoids, verbenalin (**3**), 3,4-dihydroverbenalin (**4**), hastatoside (**5**) by means of various column chromatography steps. The structures of these compounds were elucidated through analysis of their spectroscopic data obtained using 1D and 2D NMR and MS techniques. Verbeofflin I (**1**) is the new class of secoiridoid in the family Verbenaceae.

## 1. Introduction

*Verbena officinalis* L. is a medicinal herb widely distributed in the southern part of the Yellow River in China. It is used as a Chinese folk medicine for the treatment of rheumatism and bronchitis [1]. Several investigations showed that *Verbena* species contained flavonoids, terpenoids, phenyl propanoids and iridoids [2,3,4,5,6]. Iridoids isolated from *Verbena* species, for example, verbenalin and hastatoside, are characteristic constituents of *V.* *officinalis*, and they exhibit various biological activities including sleep-promoting [7], antioxidant [8] and hepatoprotective activity [9]. Moreover, the relative contents of verbenalin and hastatoside are higher than those of other characteristic iridoids in Herba Verbenae [10]. The two compounds are very fit as target constituents for quality control of Herba verbenae, so we have established a method of quality control of Herba Verbenae [10]. As a part of our ongoing study on quality control of the plant, we investigated the constituents of Herba verbenae, the present study dealt with the isolation and structural elucidation of two new iridoids, verbeofflin I (**1**) and 7-hydroxydehydrohastatoside (**2**), together with three known iridoids, verbenalin (**3**) [11,12], 3,4-dihydroverbenalin (**4**) [13], and hastatoside (**5**) [11,12,14]. To the best of our knowledge, compound **1** is the new class of secoiridoid. The structural characteristic of compound **1** is that the iridoid skeleton ring is opened between C-5 and C-6.

## 2. Results and Discussion

**Figure 1:**
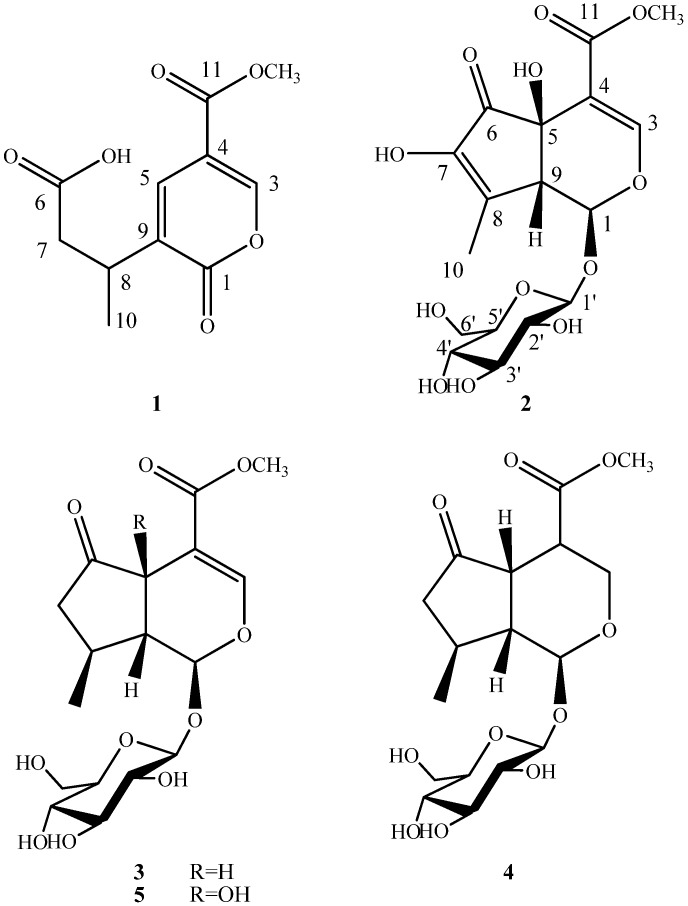
Structures of compounds **1**–**5**.

The methanol extract of *V. officialis* was stirred with diatomite and silica gel, and then extracted separately with petroleum ether, ethyl acetate and methanol. The ethyl acetate fraction was purified using a succession of silica gel and Sephadex LH-20 column chromatography and reversed-phase HPLC (C-18) to give a new iridoid (**1**) and a new iridoid glucoside (**2**), along with three known iridoid glucosides **3**–**5**. The structures of compounds **1**–**5** are illustrated in Figure 1.

**Figure 2:**
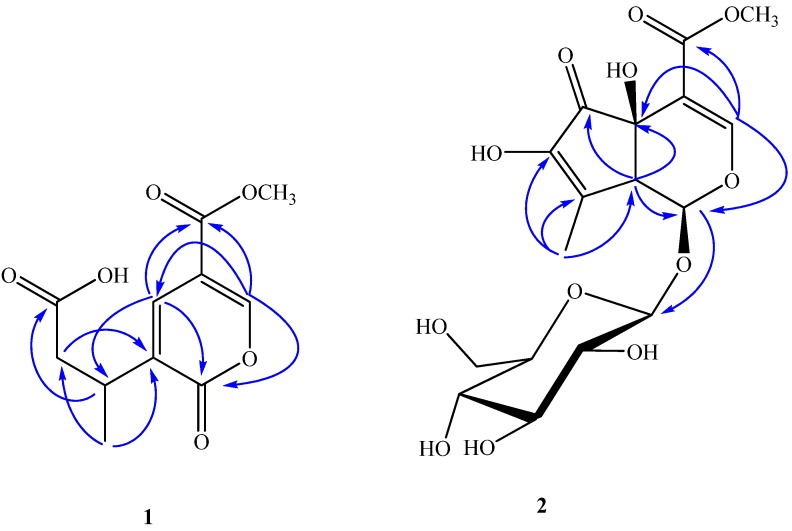
The key HMBC (H**→**C) correlations of compounds **1** and **2**.

Compound **1** was obtained as brown amorphous powder. It gave a negative result for normal chromogenic agents like H_2_SO_4_/EtOH, H_3_[P(Mo_3_O_10_)]_4_/EtOH and I_2_. It had a dark spot at λ_254__ nm_ in the UV. Compound **1** showed in its HR-ESI-MS a [M+Na]^+^ molecular ion at *m/z* 263.0530, compatible with the molecular formula C_11_H_12_O_6_Na, suggesting six degrees of unsaturation. In the analysis of the ^1^H-NMR (Table 1) and HSQC spectrum, the signals of one olefinic protons (*δ*_H_ 8.68, *d*, *J* = 0.5 Hz, H-3), one methoxyl group (*δ*_H_ 3.83, s, -OCH_3_), one methyl group (*δ*_H_ 1.25, *d*, *J* = 7.0 Hz, H-10), one methine proton (*δ*_H_ 1.25, *m*, H-8), along with protons of one methylene group (*δ*_H_ 2.71, *dd*, *J* = 6.5, 15.5 Hz, H-7a; 2.48, *dd*, *J* = 8.0, 15.5 Hz, H-7b) indicate compound **1** has an iridoid skeleton (Figure 1) [15], and this was supported by the ^13^C-NMR spectrum (10 carbon atoms in total excluding the methoxyl group (Table 1). The ^13^C-NMR spectrum of **1** showed three carbonyl c-atoms (*δ*_C_ 176.2, C-1; 175.9, C-6; 165.0, C-11), and by HMBC (Figure 2), the signal at *δ*_C_ 176.2 could be assigned to C-1 due to correlations with H-3 and H-5. Also, the second signal at low field (*δ*_C_ 175.2), could be shown to arise from C-6 from the HMBC spectrum, since a long-range connectivity ^3^*J* was observed between H-8 and C-6. The presence of only two doublets at *δ*_H_ 8.68 (*d*, *J* = 0.5 Hz, H-3) and 8.00 (*d*,*J* = 0.5 Hz, H-5) in ^1^H-NMR, and the corresponding ^13^C-NMR signals at *δ*_C_ 154.7, 135.8 indicated that C-5 and C-9 possessed a double bond. This was also confirmed by the degrees of unsaturation and HMBC spectrum, H-5 showed cross-peaks with C-1, C-3, C-8 and C-11. Furthermore, H-3 and H-8 had correlation with C-5. From the above information, compound **1** was further identified as a secoiridoid, which could be formed by ring-opening between C-5 and C-6 of the iridoid skeleton of hastatoside (**5**). Therefore, the stereochemistry at C-8 is most likely the same as that of 5. However, this needs to be confirmed by other data. From these data, thus, the structure of **1** was established as 3-(5-(methoxycarbonyl)-2-oxo-2*H*-pyran-3-yl)butanoic acid, named verbeofflin I. 

**Table 1:** The NMR spectroscopic data of compounds **1** and **2** (MeOH-d_4_, ^1^H-NMR 500 MHz, ^13^C**-**NMR 125 MHz).

Position	1		2	
	^1^H	^13^C	^1^H	^13^C
1	-	176.2	5.94 (1H, *d*, *J* = 1.5 Hz)	94.1
3	8.68 (1H, *d*, *J* = 0.5 Hz)	163.5	7.37 (*s*)	154.8
4	-	120.9	-	109.7
5	8.00 (1H, *d*. *J* = 0.5 Hz)	154.7	-	71.7
6	-	175.9	-	198.4
7	2.71 (1H, *dd*, *J* = 6.5, 15.5 Hz)2.48 (1H, *dd*, *J* = 8.0, 15.5 Hz)	40.0	-	149.3
8	3.26 (1H, *m*)	29.6	-	137.2
9	-	135.8	3.20 (1H, like *t*, *J* = 1.5 Hz)	53.7
10	1.25 (3H, *d*, *J* = 7.0 Hz)	19.2	1.91 (3H, *d*, *J* = 1.5 Hz)	11.9
11	-	165.0	-	167.8
1ꞌ			4.45 (1H, *d*, *J* = 8.0 Hz)	100.2
2ꞌ			3.00–3.28 (1H, *m*)	71.9
3ꞌ			3.00–3.28 (1H, *m*)	77.8
4ꞌ			3.00–3.28 (1H, *m*)	74.7
5ꞌ			3.00–3.28 (1H, *m*)	78.9
6ꞌ			3.00–3.28 (1H, *m*)3.56 (1H, *dd*, *J* = 6.5, 12 Hz)	63.1
OCH_3_	3.83 (3H, *s*)	52.9	3.61 (3H, *s*)	52.1

Compound **2** was obtained as a white amorphous powder, 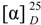
= −94.5° (*c* = 0.012, MeOH), with a *quasi*-molecular ion peak at *m/z* 441.1001 [M+Na]^+^, indicating that the molecular formula of **2** was putatively C_17_H_22_O_12_, which was further confirmed by its ^13^C-NMR spectrum (Table 1). The UV absorption maximum at 238 nm suggested that **2** had a conjugated double bond and an enol ether, which are characteristic of the -OCO-C=CH-O- chromophore found in iridoids [15]. The ^1^H-NMR and ^13^C-NMR spectra of compound **2** showed similar features to those of hastatoside [12]. The differences between the ^1^H-NMR data of **2** and that of hastatoside were signals at *δ*_H_ 1.91 (3H, *d*, *J* = 1.5 Hz, H-10), 3.20 (1H, like *t*, *J =* 1.5 Hz, H-9), and the differences between the ^13^C-NMR data of **2** and that of hastatoside were signals at *δ*_C_ 137.2 (C-8), 149.3 (C-7), those suggested that **2** possesses a second conjugated double bond, and it can be assigned at C-7-C-8, it was confirmed by the change of chemical shifts of C-6 of compound **2** showed the remarkable upfield shifts by 18ppm, compared to that of hastatoside (*δ*_C_ 215), and the HMBC spectrum analysis (Figure 2) displayed the correlation peaks between H-10 and C-7. Furthermore, in HMBC spectrum, the olefinic proton at *δ*_H_ 7.37 (H-3) showed cross-peaks with C-11, C-5, and C-1, which suggested that the attachment hydroxyl group was assigned to C-5; A methine proton at *δ*_H_ 3.20 (1H, *t*, *J* *=* 1.5 Hz) correlated with C-4, 6, 7 and 10, which implied that the proton should be assigned at C-9; the anomeric proton at *δ*_H_ 4.45 (1H, *d*, *J* *=* 8.0 Hz) showed correlations with C-1, which suggested that the sugar moiety was located at C-1. The attachment of the hydroxyl group was assigned to C-7 due to the analysis of the molecular formula. In the meantime, it was proved by the ^1^H-NMR spectrum. In the ^1^H-NMR, there wasn’t another olefinic proton except for H-3. Confirmation of the stereochemistry of its stereogenic centers was achieved by analysis of *J* values and comparison with ^13^C-NMR literature data, especially those for chiral centers at C-1, C-5, and C-9, which indicated the β configuration of the hydroxyl group at C-5, consistent with the configuration of this substituent in hastatoside, The *cis* junction between the two rings and the *O*-glycosyl residue at C-1 is biosynthetically supported with a β-configuration [16]. From the above information, compound **2** was thus identified as 7-hydroxydehydrohastatoside.

## 3. Experimental

### 3.1. Plant Materials

The aerial part of *V. officinalis* was collected in Chongqing, China, and was identified by Prof. Guoyue Zhong of The Institute of Chinese Materia Medica of Chongqing. A voucher specimen (No. 20070507) was deposited in Shanghai R&D Center for Standardization of Chinese Medicines. 

### 3.2. General Procedure and Regents

^1^H (500 MHz), ^13^C (125 MHz), and 2D NMR spectra were obtained on Bruker AV-500 (Bruker Co., Fällanden, Switzerland) instrument with TMS as internal reference, using methanol-*d*_4_ as solvent. Electrospray ionisation (ESI) mass spectra were acquired in the positive and negative ion mode on a LCQ DECAXP instrument (Thermo Finnigan, San Jose, CA, USA) equipped with an ion trap mass analyzer. HR-ESI-MS were obtained in the positive ion mode on Waters UPLC Premir Q-TOF (Waters Co., Milford, CT, USA). IR spectra (expressed in in cm^−1^) were obtained on a Nicolet^TM^-380 spectrometer from Thermo Electron. Melting points were measured on a Büchi-Meting-Point-B-540 apparatus (Büchi Co., Flawil, Switzerland), and are uncorrected. Optical rotations were acquired on a Krüss-P800-T polarimeter (Krüss, Co., Hamburg, Germany). TLC plates were HSGF254 SiO_2_ from Yantai Jiangyou Silica Gel Development Co., Ltd., Yantai, China. Column chromatography (CC) silica gel (SiO_2_; 200–300 mesh; Qingdao Haiyang Chemical Co., Ltd., Qingdao, China), Sephadex LH-20 (GE-Healthcare Bio-Sciences AB, Uppsala, Sweden), ODS (SepaxGPC18, 40−60 m, Sepax Technologies Inc., Newark, NJ, USA) were employed as packing materials, semi-preparative HPLC (Waters 996 detector, Waters 717 autosamper, Waters 600 controller, Milford, MA, USA). All other chemicals were of analytical reagent grade.

### 3.3. Extraction and Isolation

The aerial part of *V. officialis* (7.5 kg) was percolated with 90% (*v*/*v*) aqueous methanol (200L) for 15 days at room temperature. The 90% MeOH extract was concentrated under reduced pressure to give a residue (995 g), which was stirred with diatomite and silica gel, then extracted separately with petroleum ether, ethyl acetate and methanol. The ethyl acetate extract was concentrated to yield a residue (316 g). The dried ethyl acetate extract (120 g) was subjected to silica gel column chromatography with gradient mixtures of CH_2_Cl_2_/MeOH (from 100:1 to 1:1), to yield six major Fractions (*Fr.1–6*). *Fr. 2* (13 g) was further subjected to repeated CC (silica gel, CH_2_Cl_2_/MeOH 18:1, and Sephadex LH-20 MeOH) to afford **1** (51.2 mg) and **3** (890.7 mg). *Fr. 3* (21 g) was further subjected to repeated CC (silica gel, CH_2_Cl_2_/MeOH 15:1, and Sephadex LH-20 MeOH) to afford **5** (912.5 mg). *Fr. 5* (32 g) was further subjected to CC (ODS 35% MeOH, and Sephadex LH-20 MeOH), followed by semi-prep. HPLC (MeOH/H_2_O, 15:85, 3.0 mL/min) to yield compounds **2** (8.2 mg, t_R_ 8.2 min), **4** (35.8 mg, t_R_ 21.6 min).

### 3.4. Spectra Data

*3-(5-(Methoxycarbonyl)-2-oxo-2H-pyran-3-yl)butanoic acid* (**1**): Brown amorphous powder (MeOH), mp 175–176 °C (MeOH). 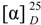 = −57.2° (*c* = 0.062, MeOH). IR (KBr, cm^−1^): 3328, 1736, 1720, 1685, 1648, 1624, 1562, 1540, 1458, 1440, 1293, 1133. ESI-MS *m/z* 241.27[M+H]^+^, 279.22[M+Na]^+^, 502.93[2M+Na]+. HRESI-MS *m/z* 263.0530 [M+Na]^+^ (C_11_H_12_O_6_Na).^ 1^H-NMR and ^13^C-NMR data see Table 1.

*7-Hydroxydehydrohastatoside* (**2**): White amorphous powder (MeOH), mp 162–164 °C (MeOH). 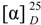
= −94.5° (c = 0.012, MeOH). IR (KBr, cm^−1^): 3318, 1733, 1722, 1652, 1628, 1560, 1535, 1448, 1288, 1074. ESI-MS *m/z* 417 [M−H]^−^, HRESI-MS *m/z* 441.1001 [M+Na]^+^ (C_17_H_22_O_12_Na). ^1^H-NMR and ^13^C-NMR data see Table 1.

## 4. Conclusions

Two new iridoids were isolated along with three known iridoids from the aerial part of *Verbena officinalis* L. Verbeofflin I (**1**) is the new class of secoiridoid in the family Verbenaceae. Biological evaluation of these compounds is under way.
